# Characteristics of dentists and patients associated with appropriate antibiotic prescriptions by French dentists: a cross-sectional study using Health Insurance databases

**DOI:** 10.1186/s12903-023-02727-3

**Published:** 2023-01-18

**Authors:** Maïa Simon, Ouarda Pereira, Marie-Louise Constant, Julie Guillet-Thibault, Céline Pulcini, Nathalie Thilly

**Affiliations:** 1 Université de Lorraine, APEMAC, Nancy, France; 2 Université de Lorraine, CHRU de Nancy, Département Méthodologie Promotion Investigation, F-54000 Nancy, France; 3 Direction Régionale du Service Médical (DRSM) Grand Est, Strasbourg, France; 4 Université de Lorraine, CHRU de Nancy, Service d’Odontologie, F-54000 Nancy, France; 5 Université de Lorraine, CHRU de Nancy, Département de Maladies Infectieuses, F-54000 Nancy, France

**Keywords:** Associated factors, Dentists, Prescriptions, Antibiotics, Appropriateness

## Abstract

**Background:**

The use of antibiotics in dental care is often unnecessary or inappropriate. Our objectives were to identify (i) Clusters of dentists grouped according to their appropriateness score based on proxy indicators’ results; and (ii) Dentists’ and patients’ characteristics associated with the appropriateness of antibiotic prescriptions.

**Methods:**

We used data of the Health Insurance reimbursement databases on antibiotics prescribed in 2019 by general dental practitioners of the Grand Est region in France. The appropriateness of antibiotic prescriptions was estimated by the results of recently published proxy indicators. We conducted a cluster analysis according to an appropriateness score calculated for each dentist, using the Ward method. We then conducted bivariate and multivariable analyses to identify characteristics associated with these clusters.

**Results:**

We included 3,014 dentists, who prescribed 373,975 antibiotics in 2019, and which were grouped into three clusters: average practices (*n* = 1,241), better (*n* = 686), and worse (*n* = 1,087) than average practices. Overall, dentists had more appropriate prescription practices when they were male (OR for belonging to cluster with “worse than average practices” = 1.37 (*p* = 0.003) for female), having a predominant surgery practice (*p* = 0.028) in the Lorraine area (*p* < 0.0001) for less years (*p* = 0.0002), when they had healthier patients (i.e., younger, with no chronic diseases, and who received less procedures), and when they had a more prudent use of drugs in general (i.e., less prescriptions of drugs, antibiotics, and non-steroidal anti-inflammatory).

**Conclusions:**

We identified clusters and characteristics associated with the appropriateness of antibiotic prescriptions made by dentists, which might help guiding antimicrobial stewardship interventions.

**Supplementary Information:**

The online version contains supplementary material available at 10.1186/s12903-023-02727-3.

## Background

Antimicrobial resistance has been listed among the ten global health issues to track in 2021 by the World Health Organisation [1]. The misuse and the overuse of antibiotics are among the main drivers of resistance [2]. Around 125,000 persons are infected by multidrug-resistant bacteria and more than 5,500 persons die of these infections each year in France [3]. In 2019, 78% of antibiotics were prescribed in the outpatient setting in France [4], with around 12% being prescribed by dentists [5].

Antimicrobial stewardship (AMS) has been defined by Dyar et al. as a “coherent set of actions which promote a responsible use of antimicrobials” [6], by reducing inappropriate and/or unnecessary antimicrobial use, in line with treatment guidelines. In 2020, the World Dental Federation (FDI) published a white paper to promote AMS in dental care. Its objective was to help dental teams to tackle antibiotic resistance by raising awareness, preventing and controlling infections, and optimising the use of antibiotics [7]. This was a welcome initiative, as several studies have shown worldwide that the use of antibiotics in dental care is often unnecessary or inappropriate [8–12], while antibiotics are only recommended in rare indications (prophylactic treatment for very specific situations or curative treatment in association with a source control procedure) [8, 13, 14].

Metrics are useful AMS tools to help optimising antibiotic use by defining targets and giving feedback to professionals and stakeholders [15]. Quality indicators need information on the clinical diagnosis to be calculated, but in most European countries including France, no computerised national systems link community drug prescriptions to clinical diagnoses [16]. To overcome this issue, our team recently developed four proxy indicators (PIs) estimating the appropriateness of antibiotic prescriptions at the dentist level, calculable without requiring information on clinical diagnoses, and using routine reimbursement databases. A cross-sectional study, conducted in two north-eastern French regions in 2019 and using these four PIs, showed that antibiotic prescriptions by primary care dentists were suboptimal and important variations between practitioners were highlighted [17].

Exploring dentists and patients’ characteristics associated with the appropriateness of antibiotic prescriptions can help guide AMS interventions by targeting specific populations. To the best of our knowledge, no study has investigated this topic yet. We previously used the same methodology to identify characteristics associated with the appropriateness of antibiotic prescriptions made by general practitioners [18].

Our objectives were to identify (i) clusters of dentists grouped according to their appropriateness score, based on PIs results; and (ii) dentists’ and patients’ characteristics associated with the appropriateness of antibiotic prescriptions.

## Methods

### Study setting and population

We considered in the present study all general dental practitioners of the Grand Est region (5,550,000 inhabitants [19]) in France (67,000,000 inhabitants), who had seen more than 100 different patients and who had prescribed more than ten antibiotics in 2019. We chose to use 2019 data because dental activity was impacted in 2020 because of the COVID-19 pandemic.

### Data source and study design

We conducted a one-year (2019) retrospective cross-sectional study using the reimbursement databases of the French Health Insurance, that covers 99% of the population. In France, all antibiotics must be prescribed by health professionals and are then reimbursed by the Health Insurance. The Health Insurance reimbursement databases can link each antibiotic dispensed by community pharmacies to the prescribers’ and patients’ characteristics such as age, gender, and the presence of chronic disease. Information on the prescription is not available, so dispensation was used as a proxy for prescription.

### Proxy indicators (PIs)

We previously developed and validated four PIs estimating the appropriateness of antibiotic prescriptions at the dentist level, calculable without information on clinical diagnoses and using routine reimbursement databases [17]. These PIs, selected from the literature and based on national guidelines [20], cover the most frequent situations of inappropriate prescribing in dental care. They were associated with a clear target reflecting a high quality of care. Performance was defined as the percentage of dentists who reached the target. Additional file 1: Table S1 presents the definition (numerator/denominator) of the four PIs and their associated target [17].

### Cluster analysis

An appropriateness score was calculated for each dentist as follows [18]:

$$\frac{{{\text{Number}}\;{\text{of}}\;{\text{PIs}}\;{\text{~applicable}}\;{\text{~for}}\;{\text{~the}}\;{\text{~dentist}}\;{\text{~and}}\;{\text{~for}}\;{\text{~whom~}}\;{\text{the}}\;{\text{~target}}\;{\text{~is}}\;{\text{~reached}}}}{{{\text{Number}}\;{\text{of}}\;{\text{PIs}}\;{\text{applicable}}\;{\text{for}}\;{\text{the}}\;{\text{dentist}}}}$$

A PI was considered applicable if it was based on at least ten situations per year for the dentist. A cluster analysis was then conducted to group dentists according to their appropriateness score, i.e., to the appropriateness of their antibiotic prescribing practices. The Ward method was used to identify clusters according to the degree of similarity between observations in a same cluster.

### Dentists’ and patients’ characteristics

The association between the appropriateness of antibiotic prescribing practices and the following 2019 dentists’ and patients’ characteristics was investigated. For each included dentist, we considered: the geographic area of professional practice (Alsace, Champagne-Ardenne or Lorraine); the dentist’s gender; the practice setting (rural (< 5,000 inhabitants), suburban (5,000 to 20,000 inhabitants) or urban (> 20,000 inhabitants)); the number of years of practice; the main type of procedure performed in daily practice (prosthetic, prevention, operative dentistry, implantology, parodontology, or surgery); the number of different patients seen during the year; the number of procedures per patient (in France, the unit of reimbursement is the procedure, which may consist in several acts and/or several visits); the percentage of patients with chronic diseases among patients seen during the year [21]; the percentage of patients with low income [22]; the percentage of patients < 16 years old; and the percentage of patients > 65 years old.

### Dentists’ drug prescribing profiles

The association between the appropriateness of antibiotic prescribing practices and the 2019 dentists’ drug prescribing profiles was explored considering the following variables: the total drugs expenses per patient; the number of units of all drugs per patient; the number of antibiotic prescriptions per 1000 patients; the number of systemic non-steroidal anti-inflammatory drugs per 1000 patients; and the number of systemic corticosteroids per 1000 patients.

### Statistical analyses

The PIs’ results were calculated and presented as medians and interquartile ranges (IQR), and performance (i.e. the percentage of dentists who reached the target) as percentage.

A hierarchical clustering method was conducted to identify clusters of dentists according to their appropriateness score, i.e. the appropriateness of their antibiotic prescribing practices. For each identified cluster, the median appropriateness score, its IQR and the performance for each PI are presented.

The dentists’ and patients’ characteristics were compared between clusters using Chi-square tests for categorical variables (expressed as percentage) and Kruskal–Wallis tests for continuous variables (expressed as medians and IQR). Characteristics associated with appropriateness of practices with a *p*-value < 0.10 in these analyses were then included in a multivariable polytomous logistic regression model to identify characteristics independently associated with the clusters with less appropriate practices as compared with the one with the most appropriate practices. When two characteristics were highly collinear, the one with the lower *p*-value in bivariate analyses was considered in the multivariable model.

For dentists’ drug prescribing profiles, only bivariate analyses (Chi-square or Kruskal–Wallis tests) were used to compare the clusters, because of the high collinearity between all considered variables. However, if appropriate practices were significantly associated with ‘total drugs expenses per patient’ and /or ‘number of units of all drugs per patient’, we planned to adjust analyses on the number of procedures per patient and the percentage of patients with chronic diseases (variables reflecting patients’ health), in order to interpret these results (i.e., to identify if the volume of drug prescriptions was due to differences in patients’ health or in dentists’ practices).

A *p*-value < 0.05 for two-sided tests was considered significant. All analyses were performed using SAS Enterprise Guide version 7.15 (SAS Institute Inc., Cary, N.C., USA).

## Results

### Characteristics of dentists and their patients

In 2019, 3,372 general dental practitioners practiced in the Grand Est region of France, and 3,014 (89.4%) met the eligibility criteria for the present study (> 100 patients and > 10 antibiotics prescribed in 2019). Their mean age was 47.5 ± 12.6 years, with a mean number of years of practice of 17.7 ± 12.6, and 56.9% were men. On average, they took care of 804 ± 391 different patients in 2019 for a mean number of 2,439 ± 1,212 procedures. The 3,014 dentists prescribed 373,975 antibiotics in 2019, that were dispensed to 308,123 different patients. Among these patients, 16.7% were aged < 16 years and 17.3% > 65 years, 11.8% had a chronic disease, and 7% had a low income.

### Appropriateness of antibiotic prescriptions

Additional file 1: Table S2 presents the results for the four proxy indicators [17]. Important variations between dentists were noted, with large interquartile ranges. Performance (percentage of dentists who reached the target) is presented in Table 1. The performance results were not optimal, particularly for the PI 4 (10.5% of dentists prescribed less than 5% of antibiotics rarely indicated in dental care) and PI 1 (39.6% of dentists prescribed at least 10 times more amoxicillin than amoxicillin-clavulanate).

**Table 1:** Description of the three clusters of antibiotic prescribing practices and performance for the four proxy indicators (*n* = 3,014 dentists)

	Global *N* = 3,014 dentists	Cluster 1 “Better than average practices” *N* = 686 dentists (22.8%) Score ^a^: 3/4 (3/4; 3/4)	Cluster 2 “Average practices” *N* = 1241 dentists (41.2%) Score ^a^: 2/4 (2/4; 2/4)	Cluster 3 “Worse than average practices” *N* = 1087 dentists (36.1%) Score ^a^: 1/4 (1/4; 1/4)
Proxy indicator (PI)	Performances (% of dentists who reached the acceptable target)			
PI 1–Amoxicillin / amoxicillin-clavulanate (ratio)*	39.6%	81.4%	38.8%	10.8%
PI 2–Estimated duration of antibiotic prescriptions (%)*	62.8%	92.4%	73.7%	31.6%
PI 3–Prescriptions of not indicated antibiotics (%)*	73.0%	96.7%	86.0%	43.3%
PI 4–Prescriptions of rarely indicated antibiotics (%)*	10.5%	31.1%	7.3%	1.1%

^a^The score is displayed with median (1st quartile; 3rd quartile) and calculated as [(number of PIs applicable for each dentist and for whom the acceptable target is reached) / (number of PIs applicable for each dentist)]

*p-value < 0.0001 for all PIs

### Cluster analysis

Three clusters of dentists were identified. Cluster 1 grouped 686 dentists (22.8%) having better than average practices, Cluster 2 grouped 1,241 dentists (41.2%) having average practices and Cluster 3 grouped 1,087 dentists (36.1%) having worse than average practices. Table 1 presents the performances of the three dentists’ clusters for each PI. Substantial differences in performances were noted between Cluster 1 and Cluster 3 (from a 30.0% difference for PI 4 to a 70.6% difference for PI 1), highlighting a huge variability in dentists’ practices.

### Dentists’ and patients’ characteristics associated with the appropriateness of antibiotic prescriptions

Table 2 presents the results of the bivariate analyses. All dentists’ characteristics were significantly associated with the appropriateness of their antibiotic prescriptions, except the practice setting. Regarding patients’ characteristics, the percentages of patients with chronic diseases and > 65 years old were significantly associated with the appropriateness of dentists’ antibiotic prescriptions.

**Table 2:** Comparison of dentists’ and patients’ characteristics between the three identified clusters–Bivariate analyses (*n* = 3,014 dentists)

Characteristics in 2019		Cluster 1 “Better than average practices” *N* = 686 dentists (22.8%)	Cluster 2 “Average practices” *N* = 1241 dentists (41.2%)	Cluster 3 “Worse than average practices” *N* = 1087 dentists (36.1%)	*p*-value
*Dentists’ characteristics*					
Geographic area %	Alsace	33.1	39.4	44.0	** < 0.0001**
	Champagne-Ardenne	14.7	18.4	24.4	
	Lorraine	52.2	42.2	31.7	
Dentist’s gender %	Male	57.8	59.3	53.6	**0.019**
	Female	42.2	40.8	46.5	
Practice setting %	Rural (< 5,000 inhabitants)	35.1	35.2	33.7	0.265
	Suburban (5,000–20,000 inhabitants)	31.5	31.1	28.7	
	Urban (> 20,000 inhabitants)	33.4	33.7	37.6	
Type of procedure ^a^ %	Prosthetic	3.2	3.9	4.3	**0.028**
	Prevention	45.5	46.2	49.7	
	Operative dentistry	48.5	48.6	45.0	
	Surgery	2.8	1.4	1.0	
Number of years of practice Median (1st quartile; 3rd quartile)		14 (3; 29)	16 (6; 29)	18 (8; 30)	** < 0.0001**
Number of patients Median (1st quartile; 3rd quartile)		744 (504; 992)	791 (557; 1025)	720 (512; 1018)	**0.001**
Number of procedures per patient Median (1st quartile; 3rd quartile)		2.9 (2.6; 3.3)	3.0 (2.7; 3.4)	3.0 (2.6; 3.5)	**0.004**
*Patients’ characteristics*					
% of patients with chronic diseases Median (1st quartile; 3rd quartile)		11.3 (9.3; 13.5)	11.9 (9.9; 14.0)	11.8 (9.9; 14.2)	**0.0003**
% of patients with low income Median (1st quartile; 3rd quartile)		5.0 (2.4; 10.4)	4.9 (2.3; 9.4)	4.4 (2.1; 9.3)	0.073
% of patients < 16 years old Median (1st quartile; 3rd quartile)		15.3 (11.2; 19.5)	14.8 (11.0; 18.8)	15.0 (10.7; 18.6)	0.197
% of patients > 65 years old Median (1st quartile; 3rd quartile)		16.3 (11.5; 21.8)	17.3 (12.6; 22.4)	17.5 (12.7; 22.4)	**0.008**

In our sample, no dentist practiced a majority of implantology or parodontology in 2019

Bold indicates a significant *p*-value (*p*-value < 0.05)

^a^In France, dentists can practice several type of procedure in their daily practice (prosthetic, prevention, operative dentistry, implantology, parodontology, or surgery)

Table 3 presents the results of the multivariable polytomous logistic analyses. The geographic area and the number of procedures per patient were significantly associated with the appropriateness of antibiotic prescriptions, as well as the dentist’s gender and the number of years of practice (only for Cluster 3). The number of patients was associated with the appropriateness of antibiotic prescriptions but not linearly: better practices were associated with an intermediate number of patients.

**Table 3:** Association between the dentists’ and patients’ characteristics and the identified clusters—Multivariable polytomous logistic analyses (*n* = 3,014 dentists)

Characteristics in 2019*		Multivariable analysis			
		Cluster 2 “average practices” VS Cluster 1 “better than average practices”		Cluster 3 “worse than average practices” VS Cluster 1 “better than average practices”	
		Odds Ratio (95% confidence interval)	*p*-value	Odds Ratio (95% confidence interval)	*p*-value
Geographic area	Lorraine	1 (reference)	–	1 (reference)	–
	Alsace	1.43 (1.16; 1.77)	**0.0009**	2.15 (1.72; 2.68)	** < 0.0001**
	Champagne-Ardenne	1.55 (1.18; 2.03)	**0.002**	2.83 (2.14; 3.73)	** < 0.0001**
Dentist’s gender	Male	1 (reference)	–	1 (reference)	–
	Female	1.06 (0.87; 1.29)	0.596	1.37 (1.12; 1.69)	**0.003**
Number of years of practice ^a^		1.01 (1.00; 1.01)	0.219	1.02 (1.01; 1.03)	**0.0002**
Number of patients		1.000 (1.000; 1.001)	**0.004**	1.000 (1.000; 1.000)	0.532
Number of procedures per patient		1.16 (1.01; 1.34)	**0.041**	1.17 (1.01; 1.35)	**0.037**
% of patients with chronic diseases		1.04 (1.01; 1.07)	**0.020**	1.04 (1.01; 1.08)	**0.023**

The characteristics included in this multivariable model were the following: geographic area, dentist’s gender, number of years of practice, number of patients, number of procedures per patient, percentage of patients with chronic diseases and percentage of patients with low income. The percentage of patients > 65 years old was not included because of high collinearity with the percentage of patients with chronic diseases (r = 0.74). The type of procedure was not included because of high collinearity with the number of procedures per patient (*p* < 0.001). The variable percentage of patients with low income was removed from the model because of its non-significance (*p* = 0.824). The reference class is Cluster 1 “better than average practices”

Bold indicates a significant *p*-value (*p*-value < 0.05)

^a^Interpretation of results: probability of belonging to cluster 2 (3) multiplied by 1.01 (1.02) as compared to cluster 1 for each additional year of practice

Overall, dentists who had the most appropriate antibiotic prescriptions were male with less years of practice, practicing in the Lorraine region, having a predominant surgery practice, with a lower percentage of patients aged > 65 years old, a lower percentage of patients with chronic diseases, and fewer procedures per patient.

### Dentists’ drug prescribing profiles associated with the appropriateness of antibiotic prescriptions

All drug prescribing variables were significantly associated with the appropriateness of antibiotic prescriptions, except the number of systemic corticosteroids prescriptions per 1000 patients (Table 4). The associations between the appropriateness of antibiotic prescriptions and the two variables, the total drug expenses per patient and the number of units of all drugs per patient, were not modified when adjusted for patients’ health (i.e., the number of procedures per patient and the percentage of patients with chronic diseases).

**Table 4:** Comparison of dentists’ drug prescribing profile between the three identified clusters—Bivariate analyses (*n* = 3,014 dentists)

Characteristics in 2019	Cluster 1 “Better than average practices” *N* = 686 dentists (22.8%)	Cluster 2 “Average practices” *N* = 1241 dentists (41.2%)	Cluster 3 “Worse than average practices” *N* = 1087 dentists (36.1%)	*p*-value
Total drug expenses (€) per patient Median (1st quartile; 3rd quartile)	9.0 (7.5; 11.0)	9.9 (8.1; 12.0)	10.9 (8.9; 13.0)	** < 0.0001**^a^
Number of units of all drugs per patient Median (1st quartile; 3rd quartile)	3.6 (2.9; 4.4)	3.7 (3.0; 4.6)	3.9 (3.2; 4.7)	** < 0.0001**^a^
Number of antibiotic prescriptions per 1000 patients Median (1st quartile; 3rd quartile)	125.6 (83.0; 182.9)	143.4 (101.2; 201.1)	133.4 (91.5; 191.7)	** < 0.0001**
Number of systemic non-steroidal anti-inflammatory prescriptions per 1000 patients Median (1st quartile; 3rd quartile)	22.0 (6.7; 50.0)	28.3 (9.7; 60.3)	25.0 (9.3; 57.9)	**0.0005**
Number of systemic corticosteroids prescriptions per 1000 patients Median (1st quartile; 3rd quartile)	0.0 (0.0; 8.4)	0.9 (0.0; 7.5)	0.9 (0.0; 6.5)	0.724

Bold indicates a significant *p*-value (*p*-value < 0.05)

^a^Same OR and *p*-values when adjusted for patients’ health (i.e., the number of procedures per patient and the percentage of patients with chronic diseases)

Overall, dentists who had the most appropriate antibiotic prescriptions prescribed less drugs, less antibiotics, less systemic non-steroidal anti-inflammatory per patient, and had less drug expenses per patient.

## Discussion

### Summary of the principal findings

The present cross-sectional study allowed us to identify three clusters of dentists according to the appropriateness of their antibiotic prescription practices: 23% of our sample of 3,014 dentists belonged to the “better than average practices” cluster, 41% to the “average practices” cluster and 36% to the “worse than average practices” one. Performance results showed that dentists’ practices were suboptimal and highly variable between dentists and between PIs.

We identified three types of characteristics that were associated with the appropriateness of antibiotic prescriptions. The first one was characteristics related to dentists. Male dentists in the Lorraine region, with less years of practice, and having a predominant surgery practice, had the best antibiotics’ prescription practices. As it has been shown for general practitioners [23], we can hypothesise that male dentists might have a better attendance to postgraduate training, which has a positive impact on knowledge [24, 25], and thus on compliance with guidelines. But this hypothesis would need further investigation. The better practices observed for the Lorraine area might be due to the differences in the training provided between the local universities (one for each area: Alsace, Champagne-Ardenne and Lorraine), and/or to the presence of the regional antimicrobial stewardship network AntibioEst [26] which started in Lorraine in 2003, before being extended to the whole Grand Est region in 2018. This regional network coordinates a multifaceted AMS strategy, including for example postgraduate training, guidelines, or infectious diseases advice freely available by phone. Previous studies in dental care have already shown that a more recent year of graduation is associated with a better level of knowledge and practice in antibiotic prescribing [24, 27]. Finally, dentists who have a predominant surgery practice could be more aware of guidelines because surgery might be at higher risk of complications than other procedures, and/or because they have less emergency as these procedures are scheduled, so their antibiotic prescriptions might be more anticipated and protocolised.

The second type of characteristics was characteristics related to patients. Dentists with more complex patients, i.e. with older patients, more patients with chronic diseases, and patients who received more procedures, were more likely to have worse than average prescription practices. As shown elsewhere [28], dentists might be more likely to prescribe antibiotics inappropriately, in particular an increased use of broad-spectrum antibiotics, to prevent a perceived increased risk of bacterial complications in these patients.

Finally, characteristics related to dentists’ drug prescribing profile were associated with antibiotics prescribing practices. Dentists with worse than average practices had higher drug expenses and prescribed more drugs, more antibiotics and more systemic non-steroidal anti-inflammatory drugs (non-steroidal anti-inflammatory drugs are usually not indicated in bacterial infections in dentistry [29]). We showed that this was not related to patients’ health but to dentists’ drug prescriptions habits. Overall, dentists who prescribed antibiotics more appropriately seemed to have an overall more prudent use of drugs, as already observed for general practitioners [18].

### Comparison with existing literature

Few published studies aimed at identifying characteristics associated with the appropriateness of antibiotic prescriptions made by dentists. Kerr et al*.* conducted in 2020 an online questionnaire-based survey to investigate potential factors that may impact the appropriateness of antibiotic prescriptions to treat adults with acute dental pain in the United Kingdom (UK). The inappropriate prescribing practice was defined as a prescription of antibiotic(s) in a clinical situation that does not require it. The authors identified that the likelihood of inappropriate prescriptions was increased by the following factors: the qualification from a non-UK university, the low or no confidence in achieving adequate local anaesthesia, the duration of the appointment (< 20 min *versus* all other times), and the lack of a postgraduate qualification [25].

A scoping review conducted in 2018 identified dentist-related factors influencing the use and the misuse of antibiotics in dentistry [14]. Some of the characteristics we identified in the present study were already reported in the literature: practice location, practice type, years in practice, and dentist’s gender.

### Strengths and limitations

This study brings innovative and useful findings, as data on the topic is scarce. However, our work has some limitations. First, we aimed to explore the association between characteristics related to dentists or their patients and the appropriateness of their antibiotic prescriptions, but we cannot prove a causal relationship. Therefore, more studies should be conducted to further investigate our results. Second, the characteristics we investigated were limited to those available from the routine reimbursement databases. We acknowledge that they do not explain all the variability of practices, and that other characteristics—not explored here—could also influence this variability.

### Implications/perspectives

The present study showed that performance results of dentists were far from optimal, especially in the clusters of dentists with average and worse than average practices, and presented a wide variability between clusters, highlighting the urgent need for AMS interventions in dentistry. Therefore, this study could be useful to help guiding AMS interventions. First, AMS interventions can target dentists belonging to the “worse than average” cluster and focus on the situations covered by PIs that showed the worst performances (e.g., prescription of amoxicillin rather than amoxicillin-clavulanate, indications for prescription of pristinamycin, spiramycin-metronidazole or doxycycline). In addition, AMS interventions can target dentists presenting characteristics that were associated with inappropriate prescriptions, such as those located in specific geographic area or those practicing for a long time. Tailoring AMS strategies and interventions to these prescribers and to situations of highest concern (i.e., frequently source of antibiotic misuse) might help to reduce the number of antibiotic prescriptions by dentists and to improve their appropriateness [30]. Consequently, AMS in dentistry might lead to an optimisation of the clinical management of infections by dentists, with an improvement of clinical results and no increase of unintended consequences (i.e., toxicity and resistance emergence) [31].

The method of the present study might also be used in other countries, by adapting the PIs and their targets to the national prescribing guidelines and exploring relevant characteristics.

## Conclusion

We identified three clusters of dentists according to the appropriateness of their antibiotic prescription practices. Performance practices were suboptimal and presented wide variations between clusters and between PIs. We identified several characteristics associated with the appropriateness of antibiotic prescriptions related to dentists or their patients that might help guiding AMS interventions.

## Supplementary Information

**Additional file 1: Supplementary Table S1.** Definition of the four PIs and their target [17]. **Supplementary Table S2.** Results for the four proxy indicators estimating the appropriateness of antibiotic prescriptions by dentists of the Grand Est region in 2019 (n = 3,014 dentists) [17].

## Data Availability

The datasets generated and analysed during the current study are not publicly available due to restrictions that apply to these data, but anonymized data is available from the corresponding author on reasonable request.

## Abbreviations

AMS: Antimicrobial stewardship
FDI: The World dental federation
IQR: Interquartile range
PI: Proxy indicator
UK: United Kingdom
