# Supplementary material for: Characteristics of dentists and patients associated with appropriate antibiotic prescriptions by French dentists: a cross-sectional study using Health Insurance databases
Source: BMC Oral Health. 2023 Jan 18;23:29. doi: 10.1186/s12903-023-02727-3 (PMC9846701; doi:10.1186/s12903-023-02727-3)
Supplement: Supplementary file 1 — Additional file 1: Supplementary Table S1. Definition of the four PIs and their target [17]. Supplementary Table S2. Results for the four proxy indicators estimating the appropriateness of antibiotic prescriptions by dentists of the Grand Est region in 2019 (n = 3,014 dentists) [17]. [file 12903_2023_2727_MOESM1_ESM.docx]

**Supplementary Table S1.** Definition of the four PIs and their target.[1]

| Proxy indicator (PI) | Numerator description | Denominator description | Target value |
| --- | --- | --- | --- |
| PI 1 – Amoxicillin/amocixillin-clavulanate (ratio) | Number of prescriptions of amoxicillin (J01CA04) | Number of prescriptions of amoxicillin-clavulanate (J01CR02) | > 10 |
| PI 2 – Estimated duration of antibiotic prescriptions (%) | Number of prescriptions > 8 days for amoxicillin (J01CA04), amoxicillin-clavulanate (J01CR02), clindamycin (J01FF01) and pristinamycin (J01FG01), and > 4 days for azithromycin (J01FA10) | Total number of prescriptions for these five antibiotics | < 10% |
| PI 3 – Prescriptions of not indicated antibiotics (%) | Number of prescriptions of lymecycline (J01AA04), minocycline (J01AA08), pivmecillinam (J01CA08), phenoxymethylpenicillin (J01CE02), cloxacillin (J01CF02), cefadroxil (J01DB05), cefuroxime (J01DC02), cefaclor (J01DC04), cefotiam (J01DC07), ceftriaxone (J01DD04), cefixime (J01DD08), cefpodoxime (J01DD13), trimethoprim-sulfamethoxazole (J01EE01), erythromycin (J01FA01), midecamycin (J01FA03), roxithromycin (J01FA06), josamycin (J01FA07), telithromycin (J01FA15), tobramycin (J01GB01), gentamicin (J01GB03), ofloxacin (J01MA01), ciprofloxacin (J01MA02), norfloxacin (J01MA06), lomefloxacin (J01MA07), levofloxacin (J01MA12), moxifloxacin (J01MA14), flumequine (J01MB07), fusidic acid (J01XC01), nitrofurantoin (J01XE01), fosfomycin (J01XX01) | Total number of antibiotic prescriptions | < 1% |
| PI 4 – Prescriptions of rarely indicated antibiotics (%) | Number of prescriptions of pristinamycin (J01FG01), spiramycin-metronidazole (J01RA04) and doxycycline (J01AA02) | Total number of antibiotic prescriptions | < 5% |

**Supplementary Table S2.** **Results for the four proxy indicators estimating the appropriateness of antibiotic prescriptions by dentists of the Grand Est region in 2019 (n = 3,014 dentists).**[1]

| Proxy indicator (PI) | Median | IQR  (1^st^ quartile ; 3^rd^ quartile) |
| --- | --- | --- |
| PI 1 – Amoxicillin / amoxicillin-clavulanate (ratio) | 7.0 | 2.5 ; 17.6 |
| PI 2 – Estimated duration of antibiotic prescriptions (%) | 5.1 | 1.1 ; 19.5 |
| PI 3 – Prescriptions of not indicated antibiotics (%) | 0.0 | 0.0 ; 1.1 |
| PI 4 – Prescriptions of rarely indicated antibiotics (%) | 14.2 | 5.3 ; 37.3 |

**Reference**

[1] Simon M, Pereira O, Guillet-Thibault J, Hulscher MEJL, Pulcini C, Thilly N. Design of proxy indicators estimating the appropriateness of antibiotics prescribed by French dentists: a cross-sectional study based on reimbursement data. Antimicrob Agents Chemother. 2021;AAC.02630-20. DOI: 10.1128/AAC.02630-20.
